# Low Blood Zinc, Iron, and Other Sociodemographic Factors Associated with Behavior Problems in Preschoolers

**DOI:** 10.3390/nu6020530

**Published:** 2014-01-27

**Authors:** Jianghong Liu, Alexandra Hanlon, Chenjuan Ma, Sophie R. Zhao, Siyuan Cao, Charlene Compher

**Affiliations:** School of Nursing, University of Pennsylvania, 418 Curie Blvd., Philadelphia, PA 19104, USA; E-Mails: alhanlon@nursing.upenn.edu (A.H.); chenjuan@nursing.upenn.edu (C.M.); sophie.r.zhao@hotmail.com (S.R.Z.); caos@sas.upenn.edu (S.C.); compherc@nursing.upenn.edu (C.C.)

**Keywords:** internalizing, externalizing, total behavior, CBCL, child, micronutrient deficiency, zinc and iron

## Abstract

Previous research supports the link among malnutrition, cognitive dysfunction, and behavioral outcomes; however, less research has focused on micronutrient deficiencies. This study investigates whether micronutrient deficiencies, specifically blood zinc and iron levels, will be associated with increased behavior problem scores, including internalizing and externalizing behaviors. 1314 Children (55% boys and 45% girls) from the Jintan Preschool Cohort in China participated in this study. Venous blood samples were collected and analyzed for zinc and iron when the children were 3–5 years old. Behavior problems were measured with the Child Behavior Checklist (CBCL), which was completed by the parents when children were in their last months of preschool (mean age 5.6 years). General linear multivariate modeling was used, with adjustment for important sociodemographic variables. The results indicate that low zinc levels alone (*p* = 0.024) and combined low zinc and iron levels (*p* = 0.022) are significantly associated with increased reports of total behavior problems. We did not find an association between low iron and behavior problems. With regards to sociodemographics, living in the suburbs is associated with increased internalizing problems, while higher mother’s education and being female were associated with decreased externalizing problems. This study suggests that micronutrient deficiencies and sociodemographic facts are associated with behavior problems in preschoolers.

## 1. Introduction

The link between early nutrition deficiency and behavior outcomes has been receiving increasing attention [1,2,3]. At the prenatal level, Neugebauer, Hoek, and Susser [4] found that the male offspring of nutritionally-deprived pregnant women had 2.5 times the normal rate of antisocial personality disorder in adulthood. At the postnatal level, in a longitudinal study from the Mauritius birth cohort [5], it was found that children with malnutrition (protein, zinc, iron and vitamin B deficiencies) at age 3 years, compared to controls, have higher externalizing behavior problems (*i.e.*, antisocial, aggressive, and hyperactive behavior) at ages 8, 11, and 17 years [6]. In another more recent longitudinal study, Galler *et al.* [3] found that children who were malnourished at an early age showed significantly higher parent-reported levels of behavior problems, particularly aggression, and decreased executive functioning at age 9–15 and again at 11–17, independent of baseline age, sex, household standard of living, and maternal depressive symptoms. Finally, at the intervention level [7], a double-blind, placebo-controlled randomized trial from England showed that supplementation of adult prisoners’ diet with vitamins, minerals, and essential fatty acids significantly reduced antisocial and violent behavior in prison. These findings have been recently replicated in young prisoners in the Netherlands [8]. This initial evidence supports the relationship between nutrition and behavioral problems; however, more research is still needed.

While increasing studies have showed the association between overall nutritional status and child behavior, few studies have specifically investigated blood zinc and iron status in relation to behavior. In developing countries, low zinc and iron levels are common [9,10,11]. Indeed, more than 90% of affected individuals live in developing countries, and approximately one-tenth of the worldwide population suffers from iron deficiency [12]. Furthermore, few studies have been conducted in Asian populations. In China, zinc and iron deficiency were previously very common, but over the past two to three decades, reports on zinc and iron intake have been mixed due to socioeconomic reform and rapid economic development that have taken place since 1979. The availability [13,14] and affordability [15] of foods have increased dramatically during this time, and as a result of this increased food production and access to food, the prevalence of malnutrition has decreased, while over-nutrition has increased [16,17]. Studies indicate that iron deficiency is less prevalent than zinc deficiency among Chinese children [18,19], and although the prevalence of anemia has decreased in China, it still exists among children [16,20]. Taken together, this makes the consideration of zinc and iron intake in Chinese samples a relevant issue for better understanding putative risk factors for behavioral outcomes.

Zinc and iron play important roles in children’s physical and behavioral health; however, there is a relative lack of attention given to the effects of specific micronutrient (e.g., zinc and iron) deficiency on behavior problems, including internalizing and externalizing disorders. Zinc is a component of enzymes that affect growth in infancy and childhood, sexual maturation, neuromotor development, and immunity. Mental function is improved by zinc’s promotion of normal brain development and physiology [21,22,23]. Iron similarly boosts mental functioning by serving as a co-enzyme involved in the production and release of neurotransmitters [21,22] and by influencing cognitive function [24,25] and behavioral disorders such as attention-deficit hyperactivity disorder [6,26].

There is now increasing evidence of the relationship between malnutrition and childhood behavior problems [27,28,29,30], though more data are needed to address the impact of specific micronutrient deficiencies on both internalizing and externalizing problems separately [2]. The importance of zinc and iron in physiological development seems to warrant particular attention with regard to how these micronutrients relate to behavioral outcomes. Childhood behavioral problems represent an important sub-area of developmental psychopathology [31,32,33,34]. Thus, identifying early childhood behavior problems—and, perhaps more importantly, their early risk factors, including nutritional and sociodemographic factors—is important for understanding and preventing problem behaviors later in life [35,36]. The purpose of this study is to assess the association of micronutrients controlled for sociodemographic factors with behavior outcomes. We hypothesize that nutritional deficiencies, specifically zinc and iron deficiencies, will be associated with increased behavior problems.

## 2. Experimental Section

### 2.1. Participants and Procedures

The current study was part of a population-based community preschool cohort study of 1656 Chinese children (55.5% boys, 44.5% girls) initially recruited between the Fall of 2004 and the Spring of 2005 from four preschools in the city of Jintan, located in the southeastern coastal region of Mainland China. In China, preschools are called kindergartens and enroll children from ages 3–6 years, after which children enter the elementary school system; to be consistent, we use preschool to refer to our study sample. Detailed sampling and research procedures of this larger cohort study have been described elsewhere [37,38]. Briefly, all children and parents taking part in the original cohort study were invited to participate for assessment of children’s behaviors while the children were in the final few months of their senior year in preschool (spring 2005 to spring 2007). At that point, some children dropped out of the study because they changed schools or because data were not fully available. Therefore, only 1385 children in the original sample were followed up in the later waves. There was no statistically significant difference between those who dropped out of the study and those who were retained [37,39].

In the last year of preschool, parents were asked to assess their children with the Chinese version of the Child Behavior Checklist (CBCL/1.5–5). Since some of the children were beyond the age limit of the CBCL/1.5–5, the current analysis only addressed the subset of the original sample that was under age 6 to adhere to the age requirement of the measure. Our final data set for analysis was thus comprised of 1314 preschoolers with a mean age of 66.6 months (SD = 5, range = 50–71), which is close to the common kindergarten age in the US. Written informed consent was obtained from parents. Institutional Review Board (IRB) approval was obtained from the University of Pennsylvania and the ethical committee for research at Jintan Hospital in China.

### 2.2. Measures

#### 2.2.1. Micronutrient Deficiency

Blood specimens were collected in Fall 2004 and Spring 2005 by trained pediatric nurses using a strict research protocol to avoid lead contamination. Approximately 0.5 mL of venous blood was collected in a lead-free EDTA tube for zinc and iron analysis. Samples were frozen and shipped to the Child Development Center, Nanjing Medical University, Nanjing, China, for analysis. Specimens remained frozen at −20 °C until analysis. Blood concentrations of zinc and iron were determined by atomic absorption spectrophotometry (BH model 5.100 manufactured by Beijing Bohu Innovative Electronic Technology Corporation), with duplicate readings taken with an integration time of 2 s. The reliability and validity of the analysis and the detailed analytic procedure have been described previously [40]. Detailed information on blood sample data collection and analysis is given in [39].

Low zinc levels were defined by concentration <76.5 μg/dL and low iron by concentration <7.5 μg/dL in blood, with cutoffs determined from the middle of the normal range. Combined low zinc and iron were defined as currently low zinc and low iron concentrations, *i.e.*, children in this category have both Zn <76.5 μg/dL and Fe <7.5 μg/dL.

#### 2.2.2. Behavior Problems at Ages 5–6

Childhood behavior problems were measured with the Chinese version of the Achenbach System of Empirically Based Assessment (ASEBA) CBCL/1.5–5 [41]. The CBCL is a widely used scale for assessing behavioral and emotional problems in children. In this study, parents were asked to answer the 99 items of the CBCL instrument, which dealt with their children’s behavior within the past 12 months, and give a rating from a 3-point scale (0 = not true; 1 = sometimes true, or 2 = often true) [41]. Factor analysis performed on the CBCL/1.5–5 has revealed two broadband factors: Internalizing behaviors and Externalizing behaviors [42]. Separately, factor analysis has produced four syndromes for Internalizing behaviors: Emotionally Reactive, Anxious/Depressed, Somatic Complaints, and Withdrawn; and two syndromes for Externalizing behaviors: Attention Problems and Aggressive Behavior [41,43]. These factor structures have also been validated in our previous study [44]. The internal reliabilities (coefficient alpha) for the scales in our study sample were as follows: Emotionally Reactive (0.71), Anxious/Depressed (0.64), Somatic Complaints (0.58), and Withdrawn (0.73), Attention Problems (0.64) and Aggression (0.87). The strategy we employ in this study is to use these established scales as predictors of latent construct “Internalizing behavior” and “Externalizing Behavior”. The sum of the items in the scales was used.

#### 2.2.3. Sociodemographic Variables

Sociodemographic information was obtained from the questionnaire filled out by the parents, and included information on gender, parental education, home living conditions, and the age of mother when the child was born. These data were collected as control variables given their potential direct effects on child behavioral problems. As discussed in our previous publication [39], we did not ask for data on household income because it is often not the best indicator of socioeconomic status, therefore we used information on house size as a proxy for evaluating socioeconomic status. A descriptive summary of these demographic variables is presented in Table 1.

**Table 1:** Baseline characteristics of study population (*N* = 1314).

Characteristic	*N*	%
**Gender**		
Male	758	55
Female	614	45
**Location**		
City	959	74
Suburb	188	14
Rural	153	12
**Mother’s Education**		
Low (≤9 years)	625	48
Medium (9–12 years)	411	32
High (>12 years)	264	20
**Family Size**		
>3 persons/household	713	57
≤3 persons/household	548	43
**House Size (m^2^)**		
<100	561	45
≥100	696	55

### 2.3. Statistical Analyses

Descriptive statistics, including frequencies and percentages, are used to characterize categorical demographic subject characteristics (Table 1). Behavioral outcomes measured on a continuum are described using means and standard deviations, and compared by low zinc and iron level groups. Multivariate modeling of behavioral outcomes regressed on low zinc and iron group, with adjustment for important sociodemographic variables (family size, gender, house size, mother’s education) is accomplished using general linear modeling. The father’s education was not included in the analysis to avoid multicolinearity because the variable was found to be highly correlated with the mother’s education. Models also accounted for clustering at the school level. Levine’s tests are used to test for homogeneity of variance across all cells. In an attempt to identify multicollinearity, bivariate associations between independent variables are examined using chi-square tests. Statistical significance was taken at the two-sided *p* < 0.05 level. All the analyses were performed using STATA version 11.0 [45].

## 3. Results

Mean behavior problem scores for total behavior, and internalizing and externalizing behavior problems are described in Table 2, Table 3 and Table 4 according to Zn level (Table 2), Fe level (Table 3), and to Zn and Fe levels (Table 4).

**Table 2:** Children’s behavior problems by Zn level (*N* = 1314).

Variable	Low Zn ^†^			Normal Zn		
	*N*	Mean	SD	*N*	Mean	SD
Total Behavior Problems	502	21.32	17.64	812	18.8	16.61
Internalizing Behavior Problems	502	6.42	5.91	812	5.74	5.84
Externalizing Behavior Problems	502	9.22	9.69	812	7.95	8.43

**^†^** Defined as zinc <76.5 μg/dL.

**Table 3:** Children’s behavior problems by Fe level (*N* = 1314).

Variable	Low Fe ^†^			Normal Fe		
	*N*	Mean	SD	*N*	Mean	SD
Total Behavior Problems	307	21.1	17.65	1007	19.36	16.85
Internalizing Behavior Problems	307	6.57	5.94	1007	5.83	5.85
Externalizing Behavior Problems	307	8.78	9.65	1007	8.33	8.73

**^† ^**Defined as iron <7.5 μg/dL.

**Table 4:** Children’s behavior problems by Zn and Fe level (*N* = 1314).

Variable	Low Zn and Fe ^†^			Others		
	*N*	Mean	SD	*N*	Mean	SD
Total Behavior Problems	215	21.41	18.2	1099	19.44	16.8
Internalizing Behavior Problems	215	6.63	5.99	1099	5.88	5.85
Externalizing Behavior Problems	215	9.07	10.18	1099	8.31	8.69

**^†^** Defined as zinc <76.5 μg/dL and iron <7.5 μg/dL.

### 3.1. Zinc Status and Children’s Behavior

Results from our analyses (Table 5) using the model of total behavior score regressed on deficient zinc showed that low zinc status was significantly associated with higher total behavior problems (*p* = 0.024) in children, along with living in the suburbs, whereas high mother’s education and being female were associated with lower behavior problems. The model of externalization score regressed on low zinc showed being female and high mother’s education are associated with significantly lower externalizing behavior scores. The model of internalizing score regressed on low zinc showed living in the suburbs to be positively associated with internalizing problems.

### 3.2. Iron Status and Children’s Behavior

We did not find significant association between low iron status and internalizing, externalizing, or total behavior problems (Table 6). However, we observed that several sociodemographic indicators were significantly associated with behavior problems. The analysis using the model of total behavior score regressed on low iron indicated that being female and having high level of mother’s education are associated with significantly lower (better) total behavior scores. Living in the suburbs was also positively associated with total behavior problems. For the model of externalization score regressed on low iron, being female and high mother’s education were also associated with significantly lower externalizing behavior scores. The model of internalizing score regressed on low iron showed living in the suburbs to be positively associated with internalizing problems.

### 3.3. Zinc and Iron Status and Children’s Behavior

Results from our analyses (Table 7) using the model of total behavior score regressed on combined low levels of zinc and iron, having low zinc and iron was significantly associated with higher total behavior problems (*p* = 0.022), whereas high mother’s education and being female were associated with reduced total behavior score. The model of externalization score regressed on combined low zinc and iron showed that being female and high mother’s education are also associated with significantly lower externalizing behavior scores. The model of internalizing score regressed on combined low zinc and iron showed living in the suburbs to be positively associated with internalizing problems.

## 4. Discussion

In this community sample of Chinese pre-school children (*N* = 1314), with micronutrient levels measured at ages 3–5 years and behavioral problems measured at mean age 5.6 years, we found an association between micronutrient deficiency and total behavior problems. Firstly, we found that low zinc concentration is positively correlated with total behavior problems. Secondly, we found that combined low blood levels of zinc and iron is positively correlated with total behavior problems. These effects remained significant after controlling for sociodemographic factors such as gender and mother’s education. We did not find a significant association between low iron status and behavior problems.

The finding of the association of zinc deficiency with child behavior problems is consistent with previous findings [46,47]. Zinc is a component of enzymes that affect growth in infancy and childhood, sexual maturation, neuro-motor development, and immunity. Specifically, zinc acts as the integral enzymatic agent in metabolic processes of proteins, carbohydrates, and lipids [48] and is used as a neurotransmitter or neuromodulator in the central nervous system [49]. Mental function is improved by zinc’s promotion of normal brain development and physiology [22,23]. A recent study reported a relationship between low zinc and greater levels of hyperactivity, anxiety, and conduct problems [50]. Indeed, animal and human models have suggested a relationship between low serum zinc and anxiety, fear-like behaviors, and depression—implicating the role of the dopaminergic and serotonergic systems [51,52].

**Table 5:** The effect of Zinc on children’s behavior problems (*N* = 1314).

Variable	Total Problems (*R*^2^ = 0.08)			Internalizing (*R*^2^ = 0.04)			Externalizing (*R*^2^ = 0.11)		
	β (SE)	95% CI	*p*	β (SE)	95% CI	*p*	β (SE)	95% CI	*p*
Low Zn ^†^	2.13 (0.33)	0.70–3.57	0.024 *	0.63 (0.31)	−0.71–1.97	0.180	1.04 (0.28)	−0.17–2.25	0.066
Gender:									
Male	-	-	-	-	-	-	-	-	-
Female	−5.27 (1.21)	−10.43–−0.05	0.049 *	−0.00 (0.54)	−2.32–2.33	0.994	−4.43 (0.38)	−6.07–−2.78	0.007 *
Location:									
City	-	-	-	-	-	-	-	-	-
Suburb	8.44 (1.47)	2.11–14.78	0.029 *	3.23 (0.32)	1.87–4.60	0.010 *	3.13 (0.91)	−0.78–7.03	0.075
Rural	1.96 (3.61)	−13.58–17.51	0.641	0.55 (0.83)	−3.03–4.13	0.576	0.96 (2.05)	−7.86–9.78	0.686
Mother’s Education:									
Low (≤9 years)	-	-	-	-	-	-	-	-	-
Medium (9–12 years)	−2.51 (1.83)	−8.44–3.41	0.209	−0.52 (0.83)	−4.07–3.04	0.595	−1.45 (0.37)	−3.05–0.15	0.060
High (>12 years)	−4.58 (0.50)	−6.71–−2.44	0.012 *	−0.74 (0.54)	−3.06–1.57	0.301	−2.79 (0.59)	−5.34–−0.26	0.042 *
Family size:									
≤3	-	-	-	-	-	-	-	-	-
>3	−0.83 (0.59)	−3.36–1.70	0.295	−0.07 (0.13)	−0.63–0.48	0.622	−0.50 (0.26)	−1.63–0.65	0.205
House Size:									
<100 m^2^	-	-	-	-	-	-	-	-	-
≥100 m^2^	−1.09 (0.91)	−5.01–2.83	0.354	−0.07 (0.14)	−0.69–0.55	0.687	−0.90 (0.46)	−2.90–1.09	0.191

^†^ Defined as zinc <76.5 μg/dL; * Statistically significant at two-sided *p* < 0.05 level.

**Table 6:** The effect of Iron on children’s behavior problems (*N* = 1314).

Variable	Total Problems (*R*^2^ = 0.07)			Internalizing (*R*^2^ = 0.04)			Externalizing (*R*^2^ = 0.10)		
	β (SE)	95% CI	*p*	β (SE)	95% CI	*p*	β (SE)	95% CI	*p*
Low Fe ^†^	1.34 (0.79)	−2.07–4.74	0.233	0.59 (0.27)	−0.59–1.77	0.166	0.31 (0.39)	−1.36–1.98	0.510
Gender:									
Male	-	-	-	-	-	-	-	-	-
Female	−5.29 (1.18)	−10.35–−0.22	0.046 *	0.00 (0.53)	−2.28–2.28	0.999	−4.43 (0.37)	−6.03–−2.84	0.007 *
Location:									
City	-	-	-	-	-	-	-	-	-
Suburb	8.61 (1.57)	1.84–15.38	0.032 *	3.27 (0.32)	1.89–4.66	0.009 *	3.22 (0.96)	−0.91–7.35	0.079
Rural	2.07 (3.75)	−14.08–18.22	0.636	0.58 (0.87)	−3.15–4.32	0.571	1.01 (2.12)	−8.11–10.14	0.680
Mother’s Education:									
Low (≤9 years)	-	-	-	-	-	-	-	-	-
Medium (9–12 years)	−2.45 (1.44)	−8.66–3.77	0.232	−0.50 (0.85)	−4.16–3.17	0.619	−1.42 (0.34)	−2.90–0.06	0.054
High (>12 years)	−4.46 (0.48)	−6.51–−2.41	0.011 *	−0.71 (0.57)	−4.88–1.59	0.336	−2.74 (0.54)	−5.08–−0.40	0.037 *
Family size:									
≤3	-	-	-	-	-	-	-	-	-
>3	−0.95 (0.55)	−3.29–1.40	0.224	−0.11 (0.12)	−0.62–0.41	0.467	−0.56 (0.24)	−1.961–0.49	0.150
House Size:									
<100 m^2^	-	-	-	-	-	-	-	-	-
≥100 m^2^	−1.23 (0.93)	−5.24–2.79	0.319	−0.11 (0.17)	−0.83–0.61	0.586	−0.97 (0.46)	−2.94–1.00	0.168

^†^ Defined as iron <7.5 μg/dL; * Statistically significant at two-sided *p* < 0.05 level.

**Table 7:** The effect of Zinc and Iron on children’s behavior problems (*N* = 1314).

Variable	Total Problems (*R*^2^ = 0.07)			Internalizing (*R*^2^ = 0.04)			Externalizing (*R*^2^ = 0.10)		
	β (SE)	95% CI	*p*	β (SE)	95% CI	*p*	β (SE)	95% CI	*p*
Low Zn & Fe ^†^	1.53 (0.23)	0.53–2.53	0.022 *	0.69 (0.23)	−0.30–1.69	0.095	0.49 (0.23)	−0.752–1.49	0.174
Gender:									
Male	-	-	-	-	-	-	-	-	-
Female	−5.27 (1.18)	−10.31–−0.21	0.046 *	−0.01 (0.53)	−2.28–2.30	0.991	−4.43 (0.37)	−6.02–−2.84	0.007 *
Location:									
City	-	-	-	-	-	-	-	-	-
Suburb	8.62 (1.58)	1.83–15.42	0.032 *	3.28 (0.32)	1.90–4.66	0.009 *	3.22 (0.95)	−0.88–7.31	0.078
Rural	2.09 (3.77)	−14.12–18.31	0.634	0.59 (0.87)	−3.14–4.32	0.564	1.02 (2.12)	−8.12–10.16	0.678
Mother’s Education:									
Low (≤9 years)	-	-	-	-	-	-	-	-	-
Medium (9–12 years)	−2.45 (1.42)	−8.58–3.67	0.227	−0.50 (0.84)	−4.12–3.13	0.615	−1.42 (0.35)	−2.91–0.07	0.054
High (>12 years)	−4.46 (0.49)	−6.57–−2.34	0.012 *	−0.71 (0.55)	−3.09–1.68	0.329	−2.73 (0.55)	−5.10–−0.37	0.038 *
Family size:									
≤3	-	-	-	-	-	-	-	-	-
>3	−0.97 (0.53)	−3.25–1.31	0.209	−0.12 (0.12)	−0.62–0.39	0.424	−0.56 (0.24)	−1.57–0.45	0.140
House Size:									
<100 m^2^	-	-	-	-	-	-	-	-	-
≥100 m^2^	−1.19 (0.94)	−5.14–2.86	0.333	−0.09 (0.17)	−0.84–0.65	0.649	−0.96 (0.46)	−2.96–1.04	0.174

^†^ Defined as zinc <76.5 μg/dL and iron <7.5 μg/dL; * Statistically significant at two-sided *p* < 0.05 level.

The association of combined zinc and iron with children’s behavior has been reported previously in [53]. It has been postulated previously that zinc and iron are thought to interact with one another for absorption. When levels of both zinc and iron are low, a more severe pattern of nutritional compromise is suggested. In fact, our results showed that blood zinc was positively correlated with blood iron (*r* = 0.31, *p* = 0.000), consistent with other published reports [54,55]. As previously discussed, zinc and iron both play important enzymatic roles in the dopamine metabolism pathways [56,57,58], and it has been suggested that zinc and iron deficiencies together can lead to additive effects in dopaminergic system alterations [50]. Interestingly, Oner *et al.* [50] also reported that while combined low serum levels of zinc and iron are correlated with increased hyperactivity, as a single nutrient effect, only zinc deficiency, not iron deficiency, was related to conduct problems and anxiety. As concluded in the paper, Oner *et al.* [50] suggested that zinc and iron deficiencies might be associated with different types of behavior problems. However, it is also possible that the association of combine low zinc and iron with behavior problems is driven by the effect of low zinc alone.

While not the focus of this current paper, the sociodemographic control variables also had effects on our outcome constructs. It is worth noting that the children whose mothers had more than 12 years education exhibited decreased externalizing behavior problems in all of our analyses. As the mothers are postulated to be the primary caregiver of the child, this observation points to the fact that children of mothers who had more education tend to exhibit less behavior problems, possibly as the result of better parenting practices in addition to better nutritional habits as results of increased education concerning nutrition. Furthermore, being female is also associated with decreased externalizing behavior and total behavior problems; such that girls in our sample tended to have lower incidents of externalizing and total behavior problems than boys.

Additionally, living in the suburbs has consistently been shown to be significantly associated with increase in total behavior problems and, even more strongly, in internalizing behavior problems. This result might not be too surprising because there has been ample evidence in the literature supporting that children of economically affluent families tend to develop more internalizing behavior problems, such as anxiety, depression, and substance abuse [59,60]. Globally, an epidemiological study has found depression rates to be higher in developed countries than in others [61]. The results from our data support the literature in that the suburban residents in our sample have the highest parental occupation and parental education levels, above both the urban and rural groups. This finding is not surprising given the suburban preschool is in a “new development zone”, where up-and-coming young parents, generally of a better educational and socio-economic background, are pursuing relatively better and higher-paid occupations. Furthermore, parents who are in the transitional stage of social and economic rise in their lives and careers are likely to have high expectation of their children. Additionally, the parents themselves live very high-pressured lives from their own occupational demands, leading to the possibility that they are more likely to suffer from internalizing problems themselves, such as anxiety and depression. Previous studies have shown that parental symptoms of depression have been associated with children’s problem behavior in clinical and community samples [62,63]. We postulate that this finding could be the result of a more stressful suburban parental lifestyle due to social and occupational stress, factors that may indirectly affect their children by not having enough time to interact with them or through a decreased emphasis on food choice, which can contribute to nutrition status. Nevertheless, future research could include stress and lifestyle factors and their effects on malnutrition.

Limitations of this study should not be overlooked. First, these findings do not establish a causal relationship between zinc, iron and behavioral disturbance. However, results from intervention trials should be considered to elucidate whether a causal relationship truly exists. The nature of the study design also required that behavior be assessed during the last year of preschool while blood micronutrient levels were assessed when the children were ages 3–5 years. Consequently, participants differed in the time between times of micronutrient and behavior assessment. Secondly, nutrition was only assessed at a single point in time, making it difficult to generalize findings and assess the role of sustained nutrition deficiency, or even nutrition deficiency during the prenatal period. As a result, we were unable to separate the effects of chronic versus acute nutrition, which may have different implications on behavior [64]. Thirdly, although we included some sociodemographic factors in our analyses, other factors, such as income, should be considered in future studies. In addition, potential confounders such as the effects of other nutrients (e.g., vitamin D), food intake, and physical activity level should also be considered. Fourthly, examination of an all-Chinese sample in this age range limits application to other cultures, as cultural, ethnic, social, and age factors impact child rearing behaviors, including nutrition and feeding. Finally, while this study only included the parent-report, it would be equally important to consider other informants. Currently, the children in our cohort are at school age, and future studies will include youth self-report of behavior to assess the relationship between micronutrient deficiencies and behavior problems.

## 5. Conclusions

Few studies have specifically examined the role of zinc and iron status in relation to child behavior. This sample of Chinese preschoolers suggests that low blood zinc is correlated to increased total behavior problems and that, additionally, combined low blood zinc and iron levels are also linked to increased total behavior problems early in childhood. Implications may include more public awareness of the importance of micronutrients. While sociodemographic factors are not easily modifiable, it is possible to encourage parents, children, and baby/child care professionals to make healthy food choices, including foods rich in zinc and iron.
